# *Helicobacter pylori* infection and inflammatory bowel disease: a crosstalk between upper and lower digestive tract

**DOI:** 10.1038/s41419-018-0982-2

**Published:** 2018-09-20

**Authors:** Yang Yu, Shengtao Zhu, Peng Li, Li Min, Shutian Zhang

**Affiliations:** Department of Gastroenterology, Beijing Friendship Hospital, Capital Medical University, National Clinical Research Center for Digestive Disease, Beijing Digestive Disease Center, Beijing Key Laboratory for Precancerous Lesion of Digestive Disease, Beijing, 100050 China

## Abstract

*Helicobacter pylori* has coexisted with humans for approximately 60,000 years and greater than 50% of the global population is infected with *H. pylori*. *H. pylori* was successfully cultured in vitro in 1983 and studies of *H. pylori* have achieved substantial advances over the last 35 years. Since then, *H. pylori* has been characterized as the primary pathogenic factor for chronic gastritis, peptic ulcer, and gastric malignancy. Numerous patients have received *H. pylori* eradication treatment, but only 1–2% of *H. pylori*-infected individuals ultimately develop gastric cancer. Recently, numerous epidemiological and basic experimental studies suggested a role for chronic *H. pylori* infection in protecting against inflammatory bowel disease (IBD) by inducing systematic immune tolerance and suppressing inflammatory responses. Here we summarize the current research progress on the association between *H. pylori* and IBD, and further describe the detailed molecular mechanism underlying *H. pylori*-induced dendritic cells (DCs) with the tolerogenic phenotype and immunosuppressive regulatory T cells (Tregs). Based on the potential protective role of *H. pylori* infection on IBD, we suggest that the interaction between *H. pylori* and the host is complicated, and *H. pylori* eradication treatment should be administered with caution, especially for children and young adults.

## Facts

- IBD etiology is mainly attributed to the complex interaction between immune dysfunction, host genetic susceptibility, and environmental factors.
- Epidemiological and basic experimental studies both suggested a protective role of chronic *H. pylori* infection against IBD.
- This protective effect on IBD could be attributed to *H. pylori*-induced systematic immune tolerance and the suppression of inflammatory response.
- Tolerogenic phenotype DCs and immunosuppressive Treg are thought to be involved in the protective mechanisms.
- Low bioactive LPS of *H. pylori* could not effectively activate NF-κB pathway and stimulate the secretion of proinflammatory factors. IL-10, TGF-β, NLRP3 inflammasome, and IL-18 are critical for the protective effect of *H. pylori* on IBD.

## Open questions

- Multicenter cohort studies revealing the status of *H. pylori* infection immediately after diagnosis of IBD is highly desirable.
- Prospective studies focusing on the pathogenesis or progression of IBD after *H. pylori* eradication therapy is urgently needed.
- The relationship between enterohepatic helicobacteria species and IBD needs to be further revealed.
- The detailed molecular mechanism underlying *H. pylori*-induced tolerogenic phenotype DCs and immunosuppressive Tregs is not yet clear.
- Considering the trade-off between gastric cancer prevention and the risk of triggering of IBD, whether an asymptomatic *H. pylori* infection should be provided with an eradication prescription is still worth discussing.

## Introduction

Inflammatory bowel disease (IBD) is characterized by chronic, nonspecific intestinal inflammation with an unexplained pathology and an alternating relapsing and remitting clinical progression. IBD is divided into two subtypes: ulcerative colitis (UC) and Crohn’s disease (CD). The pathological features of IBD include enhanced TH1 and/or TH17 responses, and dramatically increased production of inflammatory factors in mucosal lesions, including tumor necrosis factor-α, interleukin (IL)-1β, interferom (IFN)-γ, IL-17, IL-6, and IL-23^1–7^. Most studies in the IBD field attribute its etiology to the complex interactions among immune dysfunction, genetic susceptibility of the host, and environmental risk factors. Autoimmune abnormalities are now widely considered one of the causes of IBD. Most patients with IBD have an individual or family history of nodular erythema, arthritis, ophthalmic uveitis, vasculitis, or systemic lupus erythematosus. In addition, mutants in autophagy genes (ATG16L1/NOD2/IRGM) were identified as inducers of aberrant immunopathological responses and impair the mucosal barrier^8,9^. In addition, the intestinal flora is considered an indispensable factor for intestinal inflammation, as most germ-free IL-10-deficient mice never develop colitis^10–12^. Given the dramatically increased prevalence in most developing countries^9,13–20^, IBD has become a substantial global medical burden and modern refractory disease, as cited by the World Health Organization, in the last two decades ^1,13–15^.

*Helicobacter pylori* is a Gram-negative, spiral-shaped bacillus. It successfully colonizes the gastric mucosa due to its specific motility, microaerobic metabolism, and anti-acid activity^21^. *H. pylori* secretes vacuolating cytotoxin (VacA) and cytotoxin associated gene A antigen(CagA) proteins and other virulence factors to induce a TH1-dominated inflammatory response. Although Warren and Marshall^22^ first discoverd *H. pylori* in 1983, *H. pylori* has coexisted with humans for a considerably longer period. Biogeography studies cite *H. pylori* as a witness to human migration history from East Africa approximately 60,000 years ago, and paleomicrobiologists found *H. pylori* in the oldest mummies and the Alps Iceman who lived in 5200 years ago. In the early twentieth century, numerous researchers observed the spiral bacteria on gastric mucosal surface, but the existence of *H. pylori* was not confirmed until Warren and Marshall^22^ discovered it. Subsequently, researchers found almost all *H. pylori*-infected patients exhibit histological, chronic, active inflammation, even asymptomatic *H. pylori*-infected individuals^23^. In addition, the inflammatory response is reduced after *H. pylori* eradication and *H. pylori* was identified as the pathogenic factor that directly causes chronic gastritis and a class I biological carcinogenic factor in gastric cancer^24^. According to the 2015 “*Helicobacter pylori* gastritis Kyoto global consensus report”, *H. pylori* gastritis should be defined as an “infectious disease” and all *H. pylori*-positive patients should receive eradication therapy, regardless of the presence of gastric ulcers or gastric cancer^25^. However, although approximately half of the global population is infected with *H. pylori*, only 10–20% of *H. pylori*-infected individuals exhibit peptic ulcers, 1~2% develop gastric cancer, and < 1% exhibit gastric mucosa-associated lymphoid tissue lymphoma^26–28^. Moreover, consistent with “Africa enigma,” recently reported gastric cancer prevalence is also much lower in less developed Asian countries (who have high *H. pylori* infection rates range of 55–92%) than relatively developed Asian country^29^.

## Association between *H. pylori* and IBD

**Fig. 1:**
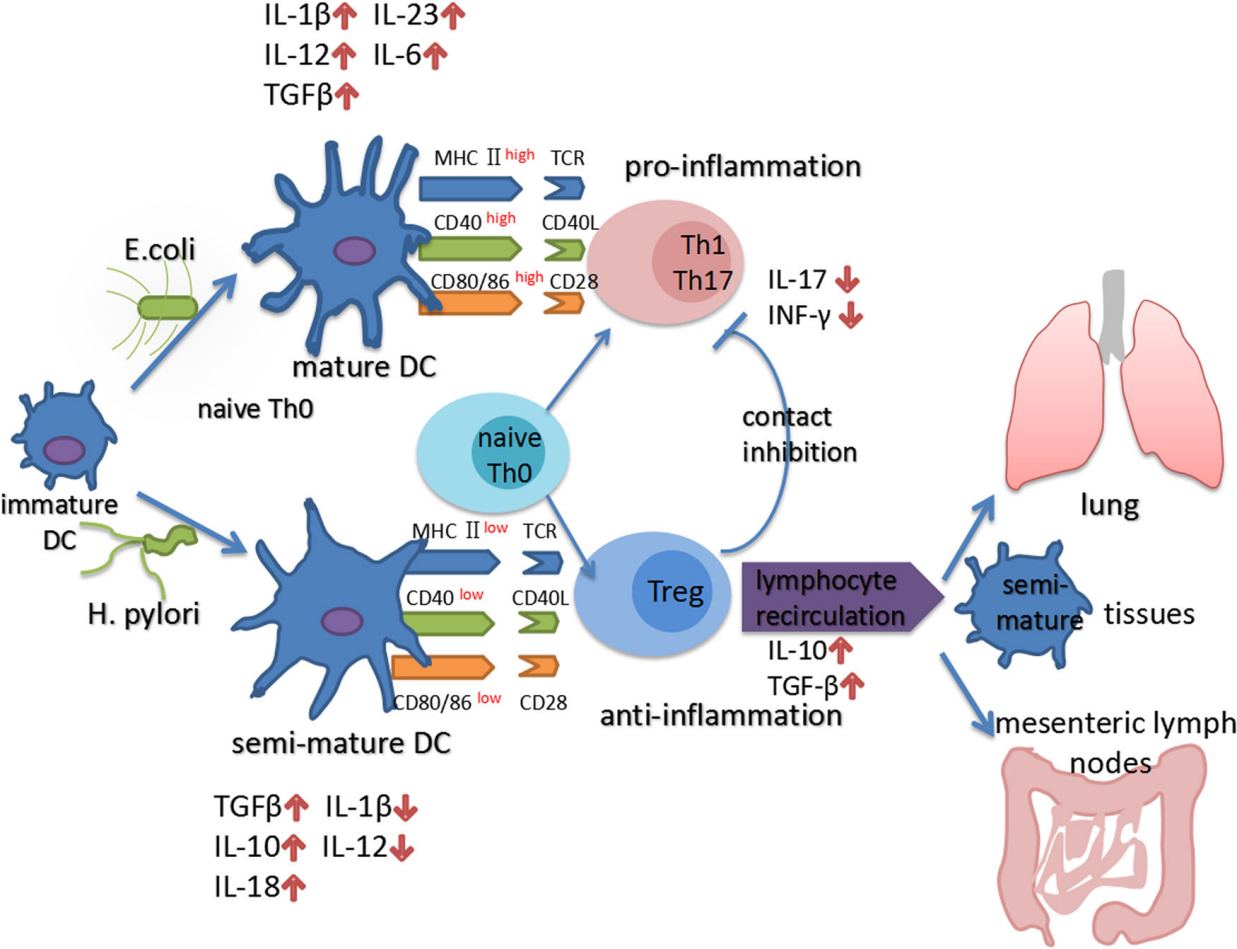
*H. pylori* infection induce tolerogenic DCs and immunosuppressive Tregs. *E. coli* efficiently promotes the transformation of DCs into mature DCs expressing high levels of MHC II CD80, CD86, and CD40that produce numerous of proinflammatory factors, such as IL-12, IL-1β, IL-6, and IL-23. In contrast, *H. pylori*-stimulated DCs retain a semi-mature phenotype with low MHC II CD80, CD86, and CD40 expression and low proinflammatory factor secretion. Semi-mature DCs secrete increased levels of IL-10, TGF-β, and IL-18, a process that is required for the differentiation of immunosuppressive Tregs, rather than Th1 or Th17 cells from naive Th0 cells. Through lymphocyte recirculation mechanisms, CD4 + CD25 + FoxP3 + Tregs produced in the gastric mucosa travel to other lymphoid tissues in distant organs to exert a systematic immunoregulatory effect that influences the pathogenesis of various autoimmune and allergic diseases, such as IBD and asthma. Moreover, Tregs inhibit the transformation of Th0 cells to Th1 and Th17 cells, and maintain DCs in a semi-mature status by direct contact and IL-10 and TGF-β secretion

**Fig. 2:**
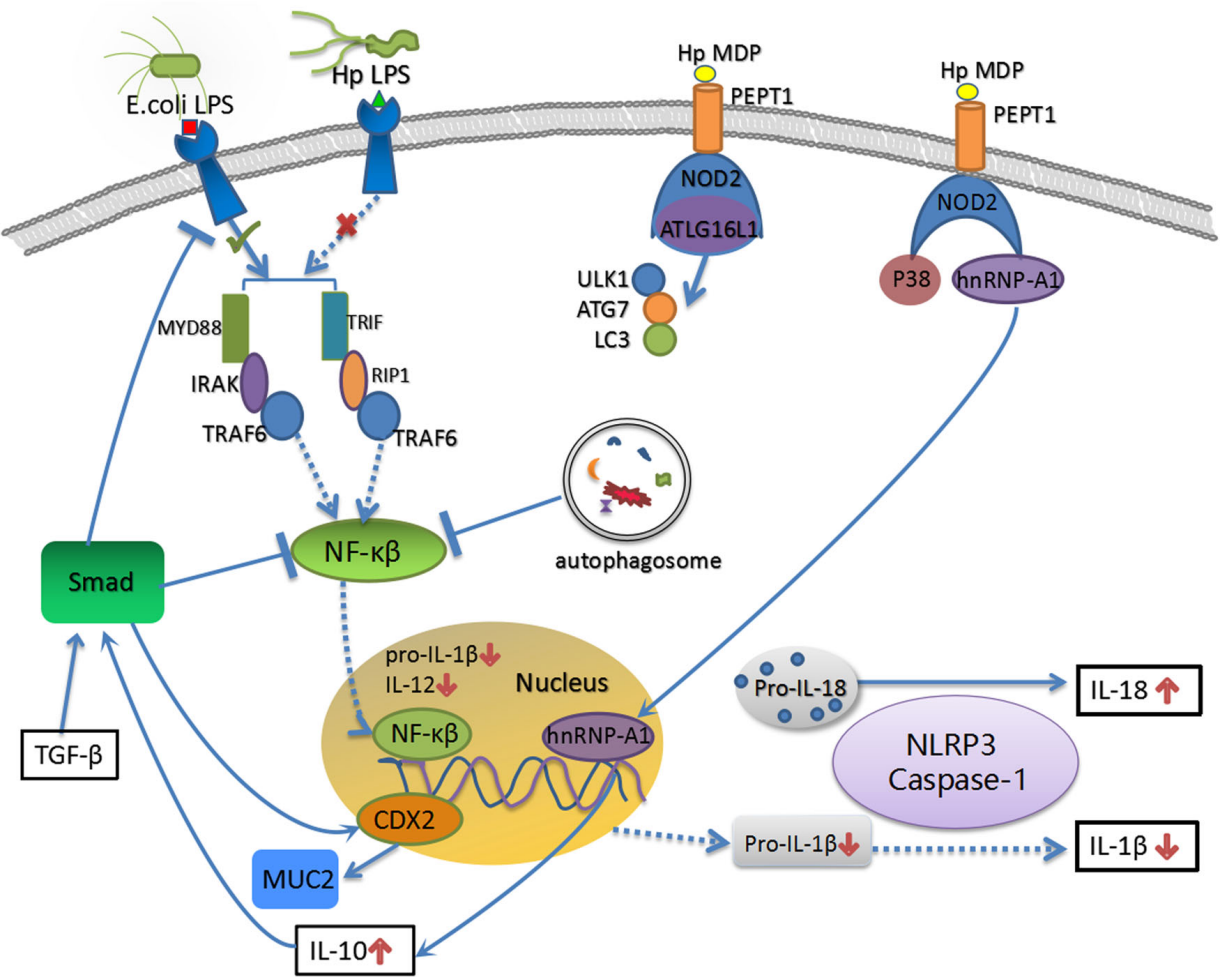
The molecular mechanism underlying the protective effect of *H. pylori* on IBD. The uncommon structure and weak biological activation of *H. pylori* LPS leads to the inefficient activation of NF-κB and production of low levels of proinflammatory molecules. On the other hand, *H. pylori* activates NOD2 and ATG16L1 to activate autophagy, and the process of autophagosome formation results in the endocytosis of MHC II and inhibition of NF-κB. The disequilibrium between inflammation and autophagy (the latter is relatively enhanced by *H. pylori* infection) may have a key role in the formation of tolerogenic semi-mature DCs. Moreover, NOD2 forms trimers with p38 and hnRNP-A1, and the latter subsequently enters the nucleus to stimulate IL-10 transcription. IL-10 and TGF-β are required for the activation of the Smad signaling pathway and downstream protective mechanisms, including the inhibition of TLR expression and the NK-κB signaling pathway and the induction of CDX2 production and MUC2 transcription. In addition, NLRP3 and IL-18 are indispensable for the protective effect of *H. pylori* on experimental colitis. Due to the NF-κB-independent production mechanism, pro-IL-18 is stably expressed in the cytoplasm and is effectively produced by activated NLRP3 and caspase-1 after *H. pylori* infection

Recently, emerging epidemiologic studies and animal experiments^30^ revealed an inverse correlation between *H. pylori* infection and IBD onset, suggesting that *H. pylori* colonization exerts a special protective effect on autoimmune diseases. Since the twenty-first century, improving hygienic conditions and socioeconomic status have reduced the *H. pylori* infection rate and this trend has concurrently been accompanied by an increased IBD incidence in most countries^31^. Most experts in the IBD field interpret this phenomenon based on the “hygiene hypothesis”: *H. pylori* infection during childhood contributes to immune system development and may prevent the onset of autoimmune or allergic diseases. Moreover, due to the initiation of *H. pylori* eradication for peptic ulcers, the incidence of IBD has increased steadily in these regions^9^. Further clarification of the protective effect of *H. pylori* on IBD and the underlying mechanism will be important for *H. pylori* infection management strategies and the treatment and prevention of IBD (Fig. 1 and Fig. 2).

### Enterohepatic helicobacteria participate in IBD pathogenesis

Various non-pylori *Helicobacter* organisms in the Helicobacteraceae family have been found to be able to colonize throughout the gastrointestinal tract and are defined as enterohepatic helicobacteria species (EHS). In addition, 16s rDNA sequencing of colonic biopsies^32,33^ and fecal samples^34,35^ revealed an increased prevalence of the Helicobacteraceae family in children with CD, particularly *Helicobacter bilis* and *Helicobacter hepaticus*. A meta-analysis^36^ further revealed an increased prevalence of EHS among patients with IBD compared with the control group (RR(relative risk) = 2.01, 95% confidence interval (CI): 1.36–2.98). In fact, some EHS have been routinely used to induce experimental colitis in immunodeficient animals^37^. In a study on the pathogenic mechanism of EHS by Kullberg et al.^38^., *H. hepaticus* infection elicited persistent colitis in IL-10^−/−^ mice by stimulating an IL-12(p35/P40)-dependent Th1 reaction. Subsequently, Kullberg et al.^39^. further verified that the IL-23(P40/P19)-dependent Th17 reaction also played a key role in an *H. hepaticus*-induced mouse colitis model. Other pathogenic mechanisms reported in related studies include disruption of the intestinal epithelial integrity by the type VI secretion system^40^, disruption of the eukaryotic cell cycle via the production of a cytolytic distending toxin^41^, and alterations in normal flora colonization to reduce flora diversity^42,43^. Based on these findings, intestinal Helicobacteraceae colonization is a potentially pathogenic factor for IBD, not a protective factor.

### The potential protective effect of *H. pylori* infection on IBD

Numerous studies have reported a lower *H. pylori* infection rate in patients with CD and/or UC than in non-IBD control individuals^44,46–49,51–55,57–66^, although a small number of studies showed no significant association^45,50,56^ (Table 1). The inverse correlation between IBD and *H. pylori* infection suggests that the gastric mucosa colonization of *H. pylori* can potentially protects against the pathogenesis of IBD via a special mechanism. Two meta-analyses^67,68^ (including 23 and 33 studies, separately) provide more powerful evidence supporting this protective effect of *H. pylori* infection on the prevalence of IBD (RR = 0.64, 95% CI: 0.54–0.75 and RR = 0.62, 95% CI: 0.55–0.71). However, the significant heterogeneity^67,68^ among the included studies and the potential publication bias^68^ largely limited the confidence of this negative correlation. Differences in *H. pylori* detection methods, IBD diagnostic criteria, study sites, participant ages, and histories of antibiotic therapy potentially contribute to the severe heterogeneity, which was not resolved by a subgroup analysis. However, a recent meta-analysis^69^ without statistical heterogeneity and publication bias also reported an inverse correlation (RR = 0.48, 95% CI: 0.43–0.54) between *H. pylori* infection and IBD prevalence in an Asian population (Table 2).

**Table 1:** Prevalence of *H. pylori* infection in patients with IBD compared with the control population

Author	IBD		NC		*χ* ^2^	*p*-Value	HP test	Control selection	Year	Country
	*H. pylori* ( + ) N (%)	*H. pylori* ( − ) N (%)	*H. pylori* ( + ) N (%)	*H. pylori* ( − ) N (%)						
Halme et al.^44^	30 (15%)	170 (85%)	43 (43%)	57 (57%)	28.3869	< 0.0001	*H. pylori* IgG ( + )	Patients with acute dysentery	1996	Finland
Pearce et al.^45^	16 (17.2%)	77 (82.8%)	10 (25%)	30 (75%)	1.0808	0.2985	UBT*	IBS	2000	UK
Sukerek et al.^46^	2 (5.3%)	36 (94.7%)	5 (13.2%)	33 (86.8%)	1.4164	0.2340	IHC* staining	NR*	2001	USA
Väre et al.^47^	67 (24%)	212 (76%)	26 (37%)	44 (63%)	4.9344	0.0263	*H. pylori* IgG ( + )	NR	2001	Finland
Matsumura et al.^48^	15 (16.7%)	75 (83.3%)	211 (40.2%)	314 (59.8%)	18.2909	< 0.0001	*H. pylori* IgG ( + )	Healthy volunteers	2001	Japan
Feeney et al.^49^	26 (9.4%)	250 (90.6%)	43 (15.6%)	233 (84.4%)	4.7864	0.0287	*H. pylori* IgG ( + )	Non-IBD patients	2002	UK
Parlak et al.^50^	74 (66.7%)	37 (33.3%)	49 (63.3%)	28 (36.7%)	0.1846	0.6675	IHC staining	Non-IBD patients	2002	Turkey
Prónai et al.^51^	17 (12.8%)	116 (87.2%)	78 (39%)	122 (61%)	26.9294	< 0.0001	UBT	Non-IBD patients	2004	Hungary
Sladek et al.^52^	9 (9.6%)	85 (90.4%)	40 (38.4%)	64 (61.6%)	22.1234	< 0.0001	UBT	Non-IBD patients	2006	Poland
Song et al.^53^	80 (25.3%)	236 (0.747%)	166 (52.5%)	150 (0.475%)	49.23	< 0.0001	UBT	Healthy volunteers	2009	Korea
Pang et al.^54^	33 (31.1%)	73 (68.9%)	65 (61.3%)	41 (38.7%)	19.4314	< 0.0001	*H. pylori* IgG ( + )	Healthy volunteers	2009	China
Li et al.^55^	13 (26%)	37 (74%)	28 (56%)	22 (44%)	9.3024	0.0023	UBT	Non-IBD patients	2010	China
Pellicano et al.^56^	12 (60%)	8 (40%)	12 (41%)	17 (59%)	1.6423	0.2000	UBT	Non-IBD patients	2010	Italy
Zhang et al.^57^	40 (19.2%)	168 (80.8%)	203 (48.8%)	213 (51.2%)	50.98	< 0.0001	UBT	Healthy volunteers	2011	China
Sonnenberg and Genta^58^	48 (4.5%)	1016 (95.5%)	5801 (9%)	58,650 (91%)	25.9461	< 0.0001	IHC staining	Healthy volunteers	2011	USA
Xiang et al.^59^	62 (27.1%)	167 (72.9%)	119 (47.9%)	129 (52.1%)	22.1069	< 0.0001	UBT	Non-IBD patients	2013	China
Jin et al.^60^	47 (30.5%)	106 (69.5%)	69 (57.0%)	52 (0.43%)	19.1521	< 0.0001	UBT	Non-IBD patients	2013	China
Xin et al.^61^	33 (18.4%)	146 (81.6%)	43 (41.3%)	61 (58.7%)	17.5774	< 0.0001	UBT	IBS	2013	China
Ali et al.^62^	6 (1.7%)	341 (98.3%)	288 (29.3%)	696 (70.7%)	23.4916	< 0.0001	IHC staining	Non-IBD patients	2013	United States
Roka et al.^63^	6 (3.8%)	153 (96.2%)	160 (13.2%)	1049 (86.8%)	11.7957	0.0006	UBT	Non-IBD patients	2014	Greece
Ma et al.^64^	38 (47.5%)	42 (52.5%)	53 (66.3%)	27 (33.7%)	5.7334	0.0166	UBT	Healthy volunteers	2016	China
Shi et al.^65^	114 (69.0%)	51 (0.31)	146 (93.9%)	9 (6.1%)	33.0582	< 0.0001	UBT	Non-IBD patients	2017	China
Zhou et al.^66^	19 (32.8%)	39 (67.2%)	53 (66.3%)	27 (33.7%)	15.1143	0.0001	UBT	Non-IBD patients	2017	China

*IBD* inflammatory bowel disease, *IHC* immunohistochemistry, *NR* not reported by Kaakoush, *UBT* urea breath test

**Table 2:** Meta-analysis of *H. pylori* infection rates in patients with IBD

Author	Subgroup	Pooled RR/OR	95% CI	*p*-Value	Heterogeneity		Publication bias
					*I* ^2^	*p*-Value	
Luther et al.^67^	IBD	0.64	0.54–0.75	NR	75.80%	< 0.001	NR
	CD	0.6	0.40–0.72	NR	NR	NR	
	UC	0.75	0.62–0.90	NR	NR	NR	
Rokkas et al.^68^	IBD	0.62	0.55–0.71	< 0.001	77%	< 0.001	0.15
	CD	0.38	0.31–0.47	< 0.001	59.50%	< 0.001	
	UC	0.53	0.42–0.67	< 0.001	62%	< 0.001	
Wu et al.^69^	IBD	0.48	0.43–0.54	< 0.001	21%	NR	0.203
	CD	0.43	0.37–0.50	< 0.001	43.00%	NR	
	UC	0.55	0.48–0.64	< 0.001	0%	NR	
Castañorodríguez et al.^74^	IBD	0.426	0.362–0.502	< 0.001	62%	< 0.001	NR
	CD	0.38	0.31–0.47	< 0.001	NR	NR	
	UC	0.53	0.44–0.65	< 0.001	NR	NR	

*CD* Crohn’s disease, *CI* confidence interval, *IBD* inflammatory bowel disease, *OR* odds ratio, *UC* ulcerative colitis

Some researchers^70–73^ attributed this inverse correlation to the complex medical therapies used by patients with IBD, including metronidazole, quinolone drugs, sulfasalazine, 5-aminosalicylic acid, corticosteroids, and immunosuppressants. The intake of these medications was considered a possible cause of the “spontaneous eradication” effect that leads to the low *H. pylori* infection rate in patients with IBD. However, this conclusion was not supported by other studies^53,54,57,74,75^, which reported that a history of taking sulfasalazine, 5-aminosalicylic acid, corticosteroids, and immunosuppressants was not a confounding factor for this inverse correlation. In addition, even if antibiotics reduce *H. pylori* infection rates in patients with IBD, the *H. pylori* infection rate remains significantly reduced in patients with IBD without a history of antibiotics use compared with healthy controls^53,54,57,74,75^. Multicenter prospective cohort studies that confirm the *H. pylori* infection status and therapy history immediately after IBD diagnosis are urgently needed, and better control of confounding factors in these studies should be implemented to achieve definitive conclusions.

Animal experiments also confirmed the negative correlation between *H. pylori* infection and IBD onset. As shown in the study by Fen et al.^76^, *H. pylori* infection significantly ameliorates colitis and histopathological changes in a DSS-induced mouse colitis model. This pathological difference is accompanied by reductions in splenic CD4 + T cells and the extent of systemic inflammation. Using mice co-infected with *H. pylori* and *Salmonella typhimurium*, Higgins PD^77^ reported that *H. pylori* inhibits the Th17 response to *S. typhimurium* infection and increases IL-10 levels in mesenteric lymph nodes. Based on the results of these studies, *H. pylori* infection affects the immune response in the lower digestive tract and involves potential immunological crosstalk between the upper and lower gastrointestinal tracts.

## *H. pylori* infection induces tolerogenic DCs

### Dendritic cells (DCs) capture *H. pylori* antigens in the gastric cavity

 Although numerous epidemiological studies and meta-analyses support the inverse correlation between *H. pylori* infection and IBD onset, the protective mechanism by which the upper digestive tract colonization of *H. pylori* can protect against IBD remains unclear. As the most powerful antigen-presenting cell and the unique activator of naive T lymphocytes (Th0), DCs have a key role in modulating adaptive immunity through the presentation of pathogen antigens and induce Th0 cells to differentiate into different lymphocyte subsets. Using two-photon microscopy to observe transgenic pCD11c-YFP mice, Kao et al.^78^ reported that CD11c + DCs are located near the gastric luminal surface and submucosal layer, and the number of DCs in the lamina propria was dramatically increased and DCs moved closer to the epithelial surface after *H. pylori* infection. Moreover, through a three-dimensional co-culture system that includes monocytes, DCs and a Caco-2 cell monolayer in a type I bovine collagen system, Leonard et al.^79^ observed DCs can move to the surface of Caco-2 cell monolayer or integrated with it. These studies indicated DCs can migrate through the intestinal epithelium to sense gastrointestinal tract antigens without impairing the integrity of the epithelial barrier.

### *H. pylori* remodel DCs to exhibit an immune tolerance property

Investigations focused on the tolerogenic property of *H. pylori*-specific DCs may help reveal the intriguing mechanism by which *H. pylori* induces systematic immunosuppression. Oertli et al.^80^ purified gastric mucosa lamina propria-derived DCs from *H. pylori*-infected patients and found that these DCs express high levels of HLA-DR and SIGN but low levels of CD80, CD83, and CD86. Kao et al.^78^ further studied the different cytokines secreted by bone marrow-derived DCs after stimulation with *H. pylori*, *Escherichia coli*, and Ruffey’s *Acinetobacter*. In this study, *H. pylori*-stimulated DCs not only maintained high transforming growth factor (TGF)-β levels but also displayed lower levels of IL-6 and IL-23 expression level than DCs stimulated with the other two positive control bacteria. IL-6 and IL-23 are important inflammatory factors that have key roles in Th17 differentiation and function maintenance^6,81–84^, suggesting that *H. pylori* has a poor pathogenicity that cannot effectively activate the inflammation pathway and Th17-modulated proinflammatory responses. This tolerogenic property also has been observed at the level of DC surface molecules. In the study by Oertli et al.^80^, prestimulate DCs with *H. pylori* in vitro significantly suppressed the *E. coli* lipopolysaccharide (LPS)-induced upregulation of CD80, CD86, and CD40. In addition, significantly lower IL-12 p40 and IL-6 levels were observed in *H. pylori*-prestimulated DCs than in the *E. coli* LPS-treated group (summarized schematically in Figure 1). Based on these evidence, although *H. pylori* infection recruits numerous DCs to the gastric mucosa, these DCs exhibit a functionally semi-mature status with an immune tolerance phenotype. This immune tolerance property of *H. pylori* may contribute to its persistent colonization of the gastric mucosa and its ability to simultaneously exert a systematic immunomodulatory effect to suppress autoimmune immunopathological responses.

### Molecular mechanism by which *H. pylori* induces tolerogenic DCs

The intrinsic nature of immune tolerance induced by *H. pylori* is attributed to the low bioactivity of its LPS. By administering intravenous injections of different LPS doses and performing three typical in vitro endotoxin tests, Muotiala et al.^85^ observed an approximately 500- to 1000-fold reduction in the biological activation of *H. pylori* LPS compared with two *Salmonella enterica* serovar *Typhimurium* subspecies (Figure 2). Long 3-hydroxy fatty acids and a deficiency of phosphorylated groups at position 4’ in the d-glucosamine disaccharide backbone of Lipid A, a constituent component of LPS, potentially explain the reduced biological activity. This uncommon structure and the significantly weaker biological activation of *H. pylori* LPS may be responsible for the formation of tolerogenic semi-mature DCs. In addition, modifications in the N-terminal TLR5 recognition domain of *H. pylori* flagellin may contribute to the escapes recognition by TLR5 (Figure 2)^86^. *H. pylori* induces DC proliferation and activates autophagosome formation in vitro^87^. *H. pylori* infection-induced autophagy activity may participate in DC remodeling process; LC3, LAMP1, and major histocompatibility complex (MHC) class II molecules were found retained in autophagic vacuoles after *H. pylori* infection; meanwhile, the surface expression of MHC II, CD80, and CD86 decreases in a TLR2/TLR4-dependent manner. Moreover, no IL-12 was detected in DCs stimulated with wild-type or VacA/CagA mutant *H. pylori* strains consistent with the downregulation of DC function and impaired T-cell proliferation (Figure 2). Based on these results, *H. pylori* infection induces TLR2/TLR4-dependent autophagy to downregulate DC function and inhibit T-cell proliferation. However, the detailed mechanism by which *H. pylori* participates in the interaction between autophagy activation and inflammatory pathways remains to be further elucidated. Moreover, some virulence factors may be necessary for the protective effect of *H. pylori* on IBD and asthma^88^. Lord et al.^89^ reported a significantly lower CagA-positive rate in patients with CD (0.94%) than in unaffected individuals (7.48%), suggesting that the CagA protein may participate in the IBD protective mechanism. Oertli et al.^90^ and Engler et al.^91^ demonstrated two dominant virulence factor γ-glutamyl transpeptidase and VacA were essential for *H. pylori*-induced tolerogenic re-programming of DCs in vivo and in vitro asthma model. However, contradictory conclusions were obtained from colitis animal model^78,80^; in these studies, the immunomodulatory effect of *H. pylori*-stimulated DCs was independent of VacA or CagA.

## *H. pylori* infection induces immunosuppressive Tregs

### Tregs participate in *H. pylori*-induced immune tolerance

As *H. pylori* strictly colonizes the gastric mucosa, the mechanism by which *H. pylori* remotely modulates lower digestive tract immune responses to influence the pathogenesis of IBD is still a subject of debate. Recently, emerging animal and in vitro experiments provided thought-provoking evidence that *H. pylori* infection of the upper digestive tract can modulate the systemic immune response by remodeling DCs to exhibit immune tolerance properties and subsequently induce Tregs polarization. Tregs are one lymphocyte subgroup that suppresses the activity of effector T cells and has a key role in maintaining immune system homeostasis and self-tolerance^92^. Forkhead box transcription factor (FOXP3) expression is required for this immunosuppressive function of Tregs. Foxp3-expressing regulatory B cell can upregulate Treg/Th17 ratio to ameliorate autoimmune arthritis^93^. Foxp3-knockout mice develop various severe or even fatal metabolic, allergic, and autoimmune diseases^94–97^. Tregs can suppress effector T-cell differentiation and proliferation by direct contact inhibition or anti-inflammatory cytokine secretion. Moreover, Tregs was shown can diminish the upregulation of costimulatory molecule on splenic DCs^98^. Tregs also participate in the pathogenesis of *H. pylori*-induced chronic gastritis and many studies report increased numbers of CD4 + CD25 + Foxp3 + Tregs in the gastric mucosa of patients with *H. pylori* infections^99,100^. Transfer of Tregs derived from *H. pylori*-pretreated neonatal mice donor attenuated ovalbumin-induced allergic airway inflammation when compared with challenged control mice^88^. Conversely, systemic Treg depletion abolished this protection effect^101^. More evidence was reported by Kao et al.^78^ ; they stimulated MACS(Magnetic Activated Cell Sorting) microbead-isolated splenic CD4 + T cells with bone marrow-derived DCs and *H. pylori* SS1 in vitro, and found that *H. pylori* induces an increased Treg ratio and decreases IL-17 levels in an IL-10- and TGF-β-dependent manner. Moreover, adoptive transfer of *H. pylori* SS1-stimulated DCs in mice induces a peripheral *H. pylori*-specific Treg response that is characterized by increased IL-10 secretion from splenic CD4 + T cells. Thus, *H. pylori*-stimulated DCs can subsequently promote Treg differentiation to induce immune tolerance.

### Tregs have a key role in systematic immunomodulation

As Tregs are required to prevent dysfunctional inflammatory responses to commensal organisms in the lower digestive tract^102^, Tregs may have a central role in chronic *H. pylori* infection-induced systematic immunomodulation and exert protective effects on IBD. This hypothesis was further verified by the effectiveness of Treg adoptive transfer therapy on mouse models of colitis or asthma^103,104^. In contrast, the dramatically reduced *H. pylori* colonization density after Treg depletion was accompanied by an enhanced peripheral Th17 response. In addition, *H. pylori*-positive patients typically present with lower peripheral type I IFN levels than the control group^105^. Based on the lymphocyte recirculation theory, we proposed that although *H. pylori* strictly colonizes the gastric mucosa, *H. pylori*-induced Tregs arrive at remote organs to suppress effector T-cell proliferation and elicit a systematic immunoregulatory effect(Figure 1). Furthermore, Onishi et al.^106^ found Tregs can aggregate around DCs and subsequently downregulate the costimulatory molecules CD80 and CD86 to maintain the semi-mature phenotype of DCs (Figure 1). Together with the lymphocyte recirculation theory, these findings explain the increase in the lung infiltration of semi-mature DCs in *H. pylori*-infected mice^101^, as *H. pylori* is unlikely to directly influence the respiratory system. In conclusion, the considerable number of Tregs induced by persistent *H. pylori* colonization in the upper digestive tract may exert a systematic immunoregulatory effect on remote organs via lymphocyte recirculation and might ultimately influence the pathogenesis of various autoimmune and allergic diseases, such as IBD and asthma.

### Molecular mechanism by which *H. pylori* induces immunosuppressive Tregs

TGF-β and IL-10 are two important and well-recognized immunoregulatory factors^107,108^, and these molecules are associated with IBD onset and Treg-modulated intestinal mucosal homeostasis^109–111^, which suggest that tolerogenic DCs may induce and maintain Treg differentiation via IL-10- and TGF-β-dependent mechanisms. Pretreatments with TGF-β- and IL-10-neutralizing antibodies reversed the ameliorated colitis pathology and the upregulation of Treg/Th17 ratio after *H. pylori* stimulation further proved this hypothesis^112,113^. Intestinal epithelial cells derived from IL-10^−/−^ mice only express RelA (p65, a phosphorylated nulcear factor (NF)-κB subunit), but not phosphorylated Smads, after pathogen stimulation^114^. Meanwhile, TGF-β was shown to activate Smad signaling to inhibit Toll-like receptor (TLR) expression and NF-κB pathway-related proinflammatory cytokine secretion^115^. In addition, Engler et al.^116^ revealed a significant correlation among CDX2, MUC2, and TGF-β, and demonstrated the activation of the TGF-β-dependent Samd-CDX2-MUC2 axis after *H. pylori* infection or extraction treatment can increases intestinal mucus secretion and ameliorate experimental colitis (summarized schematically in Figure 2). In summary, these evidences indicated TGF-β and IL-10 are critical factors for Treg differentiation and activation of protective Smad signaling after bacterial pathogen stimulation.

*H. pylori* can be successfully sensed by TLR2/NOD2 and subsequently activate NLRP3 inflammasome and caspase-1 to promote the maturation of IL-1β and IL-18^117,118^. The essential role of NLRP3 inflammasome and IL-18 for the protective effect of *H. pylori* on experimental colitis was proved by Engler et al.^116^. They found Nlrp3^−/−^, IL-18^−/−^, and IL18R^−/−^ deficient mice all lack the effective protective effect of a live *H. pylori* oral infection or intraperitoneal injection of extracts. Moreover, IL-18 was found to be required for Treg differentiation in vivo and in vitro^80^. LPS was previously shown to activate NF-κB pathway and significantly promote pro-IL-1β transcription to induce Th17 differentiation and stimulate powerful inflammatory response^119,120^. Although LPS also induces pro-IL-18 processing via the NLRP3 inflammasome, this process occurs independently of NF-κB activation due to stable storage of pro-IL-18 in cytoplasmic granules. Therefore, the inefficient perception by TLR4 and diminished NF-κB pathway due to low activity of *H. pylori* LPS lead to decreased pro-IL-1β and IL-1β levels, but not for IL-18 expression (summarized schematically in Figure 2). As IL-1β has been shown to be a strong proinflammatory cytokine^121,122^, the alterations in the relative expression levels of IL-1β and IL-18 may strikingly skew the Th1/Th17-dominated proinflammatory response to a Treg-dominated immunosuppressive response.

## The crosstalk between HP eradication and the immune response

Although *H. pylori*-associated gastroenteritis is characterized by the aggregation of local lymphocytes and polymorphonuclear cells, *H. pylori* can persistently colonize the gastric mucosa, depending on its immune escape mechanism. According to previous studies^123,124^, relatively mild gastritis in children is typically accompanied by higher levels of the Foxp3 mRNA and regulatory cytokine (IL-10 and TGF-β) expression, as well as decreased levels of the IL-17 mRNA and neutrophil infiltration in the gastric mucosa than adults with more severe gastritis. Neonatally infected mice exhibit higher density of *H. pylori* colonization due to the lack of CD4 + T-cell infiltration into the gastric mucosa. Meanwhile, neonatally infected mice derived DCs incompetently inducing Th1 effector responses from naive T cells than adult-infected group^80^. Futhermore, in a DSS-induced colitis mouse model, mice infected during neonatal period showed less pathology and less proinflammatory cytokine secretion^125^. These finding can be attributed to the different pathogenicity sense ablility and CD4 + T-cells differentiation tendency between children and adult^126^. Above evidences indicate young people whose immune system may still get remodeled can benefit more from the immune tolerance induced by *H. pylori* than older people. Another intriguing phenomenon is the significantly higher success rate of *H. pylori* clearance in patients with ulcers compared with patients with chronic gastritis. A reasonable interpretation is the immune tolerogenic property of *H. pylori*, which acquired in the long co-evolution history with human, can polarize adaptive immune to Foxp3 + Treg-dominated immunoregulatory response to favor its persistent colonization. Given the large number of Tregs and their immunosuppressive properties, patients with chronic gastritis cannot elicit a sufficiently effective immune response to eradicate *H. pylori*. However, in patients with ulcers, the breakdown of the balance between Tregs and Th1/Th17 cells transform the immune system to the latter dominating proinflammatory response, leading to more severe pathological lesions. Meanwhile, *H. pylori* is easier to eradicate using exogenous antibiotic and proton pump inhibitor treatments. Moreover, given the role of Tregs in the immune evasion strategies for some specific pathogens, Tregs depletion has been shown to elicit aggravated gastric mucosal inflammation and bacterial clearance in *H. pylori*-infected mice in vivo^103,127^.

In addition, *H. pylori* eradication therapy may trigger the onset of IBD. However, the evidence supporting this hypothesis is limited and inconclusive, because limited supportive data are available^128^. One case report from Jovanovic et al.^129^ examined one 28-year-old male patient who received 2 weeks of eradication therapy for ulcer-like dyspepsia symptoms. Six months after therapy, he experienced crampy abdominal pain, mild periodical fever, and watery diarrhea, and an endoscopic examination revealed segmental stenotic and Crohn’s-like lesions in the upper portion of the small intestines. In addition, Tursi^130^ reported two severe cases of CD (one in the terminal ileitis and one in the cecum and ascending colon) with multiple ulcers and full-thickness lymphoid infiltrates after *H. pylori* eradication therapy. The authors hypothesized that the breakdown of the equilibrium between the Th1 and Th2 responses and subsequent Th1 polarization might favor the onset of CD in some genetically susceptible individuals. However, in a small-sample *H. pylori* eradication cohort study^131^ of six patients with quiescent CD, statistically significantly differences in the CDAI (CD activity index), CRP (C-Reactive protein), and fecal calprotectin levels were not observed after *H. pylori* eradication. Further studies are urgently needed to reveal the relationship between *H. pylori* eradication and IBD onset or progression.

## Perspectives

Almost all patients with *H. pylori* infection exhibit chronic inflammation in the gastric mucosa, causing *H. pylori* to be defined as an infectious pathogen according to Koch’s law. As *H. pylori*-induced chronic atrophic gastritis is a crucial risk factor for gastric cancer, the Kyoto global consensus suggests that all *H. pylori*-infected individuals should be treated with eradication unless they present with contraindications to this treatment. Overall, eradication of *H. pylori* has not been confirmed by China’s national guidelines, considering the high infection rate and large quantities of antibiotics administered. In fact, the overall effects besides increased gastric cancer risk were largely ignored by the epidemiologists dedicated in *H. pylori* control. During the long co-evolutionary process with humans, *H. pylori* developed an immune tolerance property that favors its persistent mucosal colonization and simultaneously regulates systematic immune homeostasis by inducing tolerogenic DCs and immunosuppressive Tregs. Thus, the eradication of *H. pylori* with antibiotics not only largely influences the homeostasis of gut microbes but also has an indirect but profound effect on immune homeostasis and may lead to various autoimmune and allergic diseases, such as IBD and asthma. Just as we could not evaluate the gastric cancer risk in *H. pylori*-infected individuals accurately, we also could not perfectly evaluate the risk of IBD after *H. pylori* eradication, especially for IBD susceptible gene carriers. In conclusion, the immune tolerance property of *H. pylori* should be thoroughly considered when designing optimized and individualized treatments for *H. pylori*-infected patients.
